# DJ-1 deficiency impairs glutamate uptake into astrocytes via the regulation of flotillin-1 and caveolin-1 expression

**DOI:** 10.1038/srep28823

**Published:** 2016-06-27

**Authors:** Jin-Mo Kim, Seon-Heui Cha, Yu Ree Choi, Ilo Jou, Eun-Hye Joe, Sang Myun Park

**Affiliations:** 1 Department of Pharmacology, Ajou University School of Medicine, Suwon, Korea; 2 Chronic Inflammatory Disease Research Center, Ajou University School of Medicine, Suwon, Korea; 3 Neuroscience Graduate Program, Department of Biomedical Sciences, Ajou University School of Medicine, Suwon, Korea

## Abstract

Parkinson’s disease (PD) is a common chronic and progressive neurodegenerative disorder. Although the cause of PD is still poorly understood, mutations in many genes including *SNCA*, *parkin*, *PINK1*, *LRRK2*, and *DJ-1* have been identified in the familial forms of PD. It was recently proposed that alterations in lipid rafts may cause the neurodegeneration shown in PD. Here, we observe that DJ-1 deficiency decreased the expression of flotillin-1 (flot-1) and caveolin-1 (cav-1), the main protein components of lipid rafts, in primary astrocytes and MEF cells. As a mechanism, DJ-1 regulated flot-1 stability by direct interaction, however, decreased cav-1 expression may not be a direct effect of DJ-1, but rather as a result of decreased flot-1 expression. Dysregulation of flot-1 and cav-1 by DJ-1 deficiency caused an alteration in the cellular cholesterol level, membrane fluidity, and alteration in lipid rafts-dependent endocytosis. Moreover, DJ-1 deficiency impaired glutamate uptake into astrocytes, a major function of astrocytes in the maintenance of CNS homeostasis, by altering EAAT2 expression. This study will be helpful to understand the role of DJ-1 in the pathogenesis of PD, and the modulation of lipid rafts through the regulation of flot-1 or cav-1 may be a novel therapeutic target for PD.

Parkinson’s disease (PD) is a common chronic and progressive neurodegenerative disease. Patients with PD suffer from several motor symptoms including tremors, rigidity, hypokinesia, and difficulty with gait, which result from the death of dopaminergic neurons in the substantia nigra pas compacta (SNpc)^1^,^2^. The cause of PD is still poorly understood, however, mutations in many genes including *SNCA*, *parkin*, *PINK1*, *LRRK2*, and *DJ-1* have been identified in the familial forms of PD. The list of PD-associated genes continues to grow, which provides valuable insight into the pathogenesis of PD^3^,^4^.

DJ-1 has been identified as an early-onset, autosomal recessive gene related to PD^5^, and several familial mutations in the DJ-1 gene such as M26I, E64D, and L166P, have been reported in patients with PD^6^. DJ-1 is a ubiquitously-expressed multifunctional protein that has been implicated in several cellular processes as a molecular chaperone^7^,^8^, a transcriptional regulator^9^,^10^, and a redox sensor^11^,^12^ in a cell-specific manner. In addition, it has been reported that DJ-1 is mainly localized in the cytosol, while a small portion is localized in the mitochondria, nucleus and synaptic membrane^13^,^14^. Although the roles of DJ-1 against oxidative stress are well-known to be implicated in the pathogenesis of PD^15^, it is highly possible that DJ-1 also functions in different ways in PD, considering the variety of roles of DJ-1 in many cellular processes.

Recent evidence indicates that PD-associated genes functionally interact with each other and in common signaling pathways^16^,^17^,^18^, implying that the discovery of common signaling pathways by PD-associated genes holds great potential in the unraveling of the pathogenesis of PD. Lipid rafts are cell type-specific, variable-sized membrane microdomains that are enriched in cholesterol and glycosphingolipids, and act as platforms for the organization and interaction of proteins involved in a variety of functions, including signal transduction, receptor trafficking, and exo/endocytosis^19^. Previous reports have shown PD-associated proteins, including α-synuclein^20^, parkin^21^, PINK1^22^, LRRK2^23^, and DJ-1^24^ associating with lipid rafts, and major alterations in lipid composition have also been shown in lipid rafts purified from the frontal cortex of PD patients^25^. Considering that the sublocalization of proteins is essential for their proper functions, these observations led to the speculation that PD-associated gene products in lipid rafts may operate through common signaling pathways to develop PD, which has attracted interest recently^26^,^27^.

In our previous study, we demonstrated that DJ-1 regulates lipid raft-dependent endocytosis in astrocytes^24^. Lipid rafts-dependent endocytosis is a subgroup of heterogeneous variable endocytic pathways that is still not well-defined. Increasing evidence indicates that it includes caveolae- and flotillin- dependent endocytosis^28^,^29^. In the present study, we explore how DJ-1 regulates lipid rafts-dependent endocytosis, and whether alterations in lipid rafts as a result of DJ-1 deficiency impair glutamate uptake by astrocytes, a major function of astrocytes in the maintenance of CNS homeostasis.

## Results

### DJ-1 deficiency decreases the expression of flotillin-1 and caveolin-1

**Figure 1:**
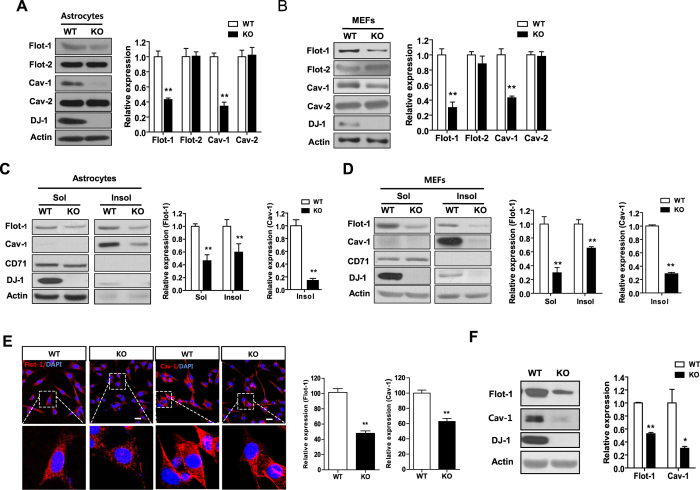
DJ-1 deficiency decreases the expression of flot-1and cav-1. Primary astrocytes (**A**) and MEF cells (**B**) from WT and DJ-1 KO mice were lysed, and the lysates were analyzed for flot-1, flot-2, cav-1, and cav-2 by Western blotting. Actin was used as a loading control. Primary astrocytes (**C**) and MEF cells (**D**) from WT and DJ-1 KO mice were lysed using ice-cold 1% Triton X-100 buffer, and fractionated as described in ‘Methods’. The soluble and insoluble fractions were analyzed for flot-1, flot-2, cav-1, and cav-2 by Western blotting. The transferrin receptor (CD71) was used as a marker for the soluble fraction. Values obtained are from three independent experiments. **p < 0.01 against WT by an unpaired t-test. (**E**) WT and DJ-1 KO MEF cells were stained with anti-flot-1 (red) or anti-cav-1 (red) antibodies, respectively. Stained cells were observed using confocal microscopy. Blue indicates DAPI staining. Scale bar indicates 20 μm. Data are representative of three independent experiments. (**F**) Whole brain lysates of WT and DJ-1 KO mice were analyzed for flot-1 and cav-1 by Western blotting. *p < 0.05, **p < 0.01 against WT by an unpaired t-test (n = 3).

To explore whether DJ-1 deficiency alters lipid rafts, we firstly examined the expression of the main protein components of lipid rafts such as flotillins and caveolins in DJ-1 knockout (KO) astrocytes. Interestingly, the expression level of flotillin-1 (flot-1) and caveolin-1 (cav-1) proteins was decreased in DJ-1 KO astrocytes compared with WT astrocytes, but the expression of flotillin-2 (flot-2) and caveolin-2 (cav-2) was not (Fig. 1A). Similar findings were observed in DJ-1 KO mouse embryonic fibroblast (MEF) cells (Fig. 1B), suggesting that alterations in flot-1 and cav-1 expression are not cell type-specific. Flotillins and caveolins are localized in lipid rafts^24^. Accordingly, in order to explore the spatial alterations in flot-1 and cav-1, lipid rafts were isolated based on their solubility in 1% Triton X-100 on ice as described previously^24^,^30^. As shown in Fig. 1C,D, the expression of flot-1 and cav-1 in the insoluble fraction of DJ-1 KO astrocytes and MEF cells was significantly decreased compared with that of the insoluble fraction of WT astrocytes and MEF cells. Additionally, the expression of flot-1 was also decreased in the soluble fraction. With respect to the confocal microscopic analysis, similar alterations in flot-1 and cav-1 expression were observed (Fig. 1E). Decreased expression of flot-1 and cav-1 was also observed in brain lysates obtained from DJ-1 KO mice, compared with those from WT mice (Fig. 1F), suggesting that DJ-1 deficiency decreases the expression of flot-1 and cav-1.

### Mutations in DJ-1 found in familial PD patients do not rescue alterations in flot-1 and cav-1 expression

**Figure 2:**
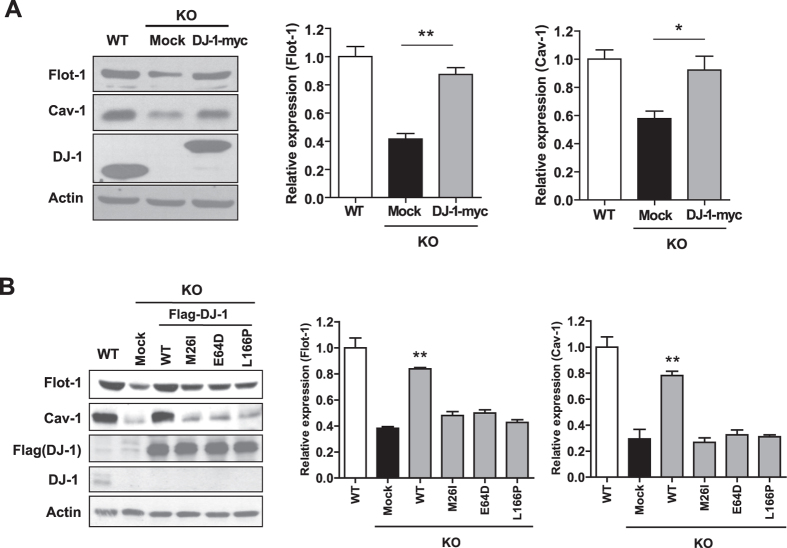
Mutations in DJ-1 found in familial PD patients do not rescue alterations in flot-1 and cav-1 expression. (**A**) WT and DJ-1 KO MEF cells were transfected with myc-tagged WT DJ-1. (**B**) WT and DJ-1 KO primary astrocytes were transfected with Flag tagged WT DJ-1 or DJ-1 mutants (M26I, E64D, and L166P). The cells were lysed and the lysates were analyzed for flot-1, and cav-1 by Western blotting. Values obtained are from three independent experiments. *p < 0.05, **p < 0.01 against mock transfectant in DJ-1 KO cells by an unpaired t-test (**A**) and one-way ANOVA (**B**).

Next, to confirm whether alterations in flot-1 and cav-1 expression in DJ-1 KO astrocytes and MEF cells are derived from DJ-1 specifically, DJ-1 was overexpressed in DJ-1 KO MEF cells. Overexpression of DJ-1 rescued the expression of flot-1 and cav-1 in DJ-1 KO MEF cells (Fig. 2A) and in primary astrocytes (Fig. 2B), confirming that alterations in flot-1 and cav-1 expression are derived from DJ-1 specifically. To further investigate whether mutations in DJ-1 found in familial PD patients affect flot-1 and cav-1 expression, three mutants of DJ-1 (M26I, E64D, and L166P) were overexpressed in DJ-1 KO astrocytes. Interestingly, these mutants did not rescue alterations in flot-1 and cav-1 expression, suggesting that the regulation of flot-1 and cav-1 expression by DJ-1 is reduced by the mutations in DJ-1 found in familial PD patients.

### DJ-1 regulates the protein stability of flot-1 and cav-1

**Figure 3:**
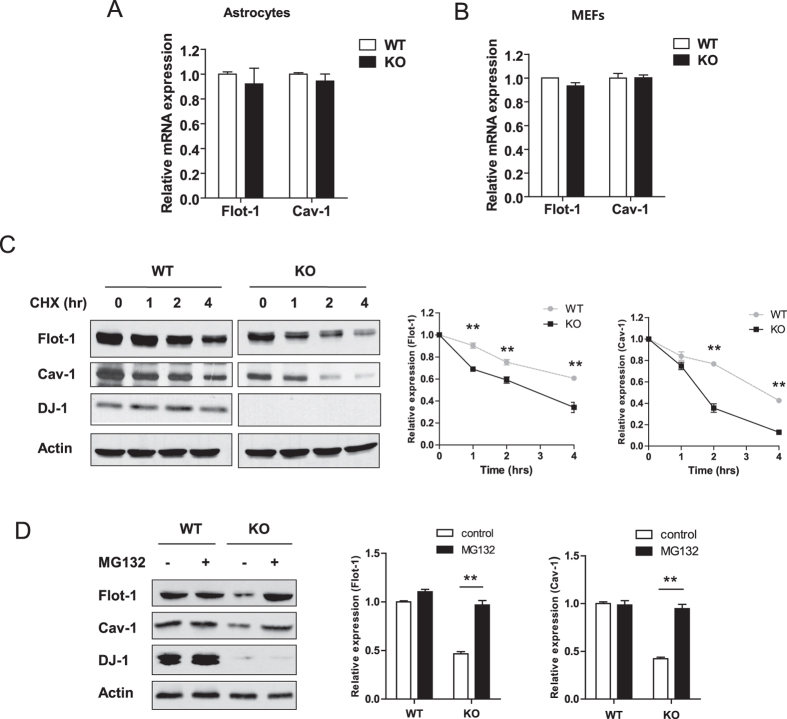
DJ-1 regulates the protein stability of flot-1 and cav-1. The mRNA level of flot-1 and cav-1 in WT and DJ-1 KO primary astrocytes (**A**) and WT and DJ-1 KO MEF cells (**B**) were analyzed by quantitative RT-PCR. The primers for actin were used as the internal control. WT and DJ-1 KO MEF cells were treated with 20 μg/ml cyclohexamide (CHX) for the indicated times (**C**), and 5 μΜ MG132 for 6 hours (**D**). The cells were then lysed and analyzed by Western blotting. Actin was used as a loading control. Values obtained are from three independent experiments. *p < 0.05, **p < 0.01 against WT by a two-way ANOVA (**C**) and against the control by an unpaired t-test (**D**).

To investigate the manner in which DJ-1 regulates the expression of flot-1 and cav-1, we firstly examined the alterations in the mRNA levels of flot-1 and cav-1 using quantitative RT-PCR. The mRNA levels of flot-1 and cav-1 were not different between WT and DJ-1 KO astrocytes (Fig. 3A) and MEF cells (Fig. 3B), implying that DJ-1 does not regulate flot-1 and cav-1 expression at the transcriptional level, and may instead regulate the protein stability of flot-1 and cav-1. To confirm this hypothesis, WT and DJ-1 KO MEF cells were treated with cycloheximide (CHX), an inhibitor of protein synthesis, and the expression of flot-1 and cav-1 was assessed using Western blotting. As shown in Fig. 3C, the degradation of flot-1 and cav-1 in DJ-1 KO MEF cells was significantly faster than in WT MEF cells. There is evidence that flot-1 and cav-1 expression is regulated by the proteasomal degradation pathway^31^,^32^. Treatment with MG132, a proteasomal inhibitor, rescued the fast degradation of flot-1 and cav-1 in DJ-1 KO MEF cells (Fig. 3D), suggesting that DJ-1 regulates the stability of flot-1 and cav-1 via the proteasomal degradation pathway.

### DJ-1 interacts with flot-1, but not with cav-1

**Figure 4:**
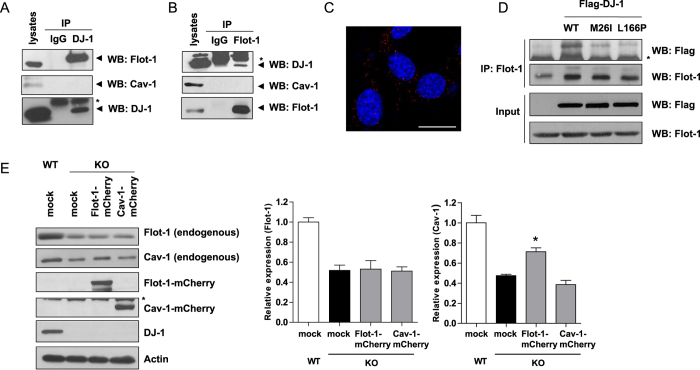
DJ-1 interacts with flot-1, but not with cav-1. (**A,B**) Lysates from WT and DJ-1 KO MEF cells were immunoprecipitated with anti-DJ-1 and anti-flot-1 antibodies, respectively and IgG as a negative control. The samples were then analyzed for DJ-1, flot-1 and cav-1 by Western blotting. Asterisk indicates Ig light chain. (**C**) An *in situ* PLA assay was performed in WT MEF cells as described in ‘Methods’. Red PLA spots represent interactions between DJ-1 and flot-1. Blue indicates DAPI staining. Scale bar indicates 10 μm. (**D**) HEK293 cells were transfected with flag-tagged WT DJ-1 and DJ-1 mutants (M26I and L166P), and were immunoprecipitated with an anti-flot-1 antibody. The samples were then analyzed for flot-1 and DJ-1 by Western blotting. Data are representative of at least three independent experiments. Asterisk indicates a non-specific band. (**E**) WT and DJ-1 KO MEF cells were transfected with flot-1-mCherry and cav-1-mCherry. After 24 hours, the cells were lysed and the lysates were analyzed for DJ-1, flot-1, and cav-1 by Western blotting. Values obtained are from three independent experiments. Asterisk indicates a non-specific band. *p < 0.05 against mock transfectant in DJ-1 KO cells by a one-way ANOVA.

Next, we investigated whether DJ-1 interacts with flot-1 and cav-1. As shown in Fig. 4A,B, DJ-1 interacted with flot-1, but not with cav-1 in WT MEF cells. The interaction between DJ-1 and flot-1 was also confirmed using an *in situ* proximity ligation assay (PLA) (Fig. 4C). In addition, when familial mutants of DJ-1 (M26I and L166P) were overexpressed in HEK293 cells, the interaction between endogenous flot-1 and DJ-1 mutants (M26I and L166P) was decreased compared with WT DJ-1 (Fig. 4D), which correlates well with the data showing that DJ-1 mutants failed to regulate flot-1 expression, as shown in Fig. 2B. Although we did not observe DJ-1 interacting with cav-1, it has been reported that down-regulation of flot-1 expression decreases the cav-1 expression level in intestinal epithelial cells^33^. Thus, to investigate whether the decrease in flot-1 expression by DJ-1 deficiency results in decreased cav-1 expression, flot-1-mCherry and cav-1-mCherry were overexpressed in DJ-1 KO MEF cells, and the expression of endogenous flot-1 and cav-1 was analyzed using Western blotting. As shown in Fig. 4E, overexpression of flot-1-mCherry rescued the decreased endogenous cav-1 expression that had been caused by DJ-1 deficiency. However, overexpression of cav-1-mCherry had no effect on endogenous flot-1 expression, suggesting that DJ-1 regulates flot-1 stability through direct interaction, and that decreased cav-1 expression may result from a decreased flot-1 level caused by DJ-1 deficiency.

### Alteration in cellular cholesterol level and membrane fluidity by DJ-1 deficiency

**Figure 5:**
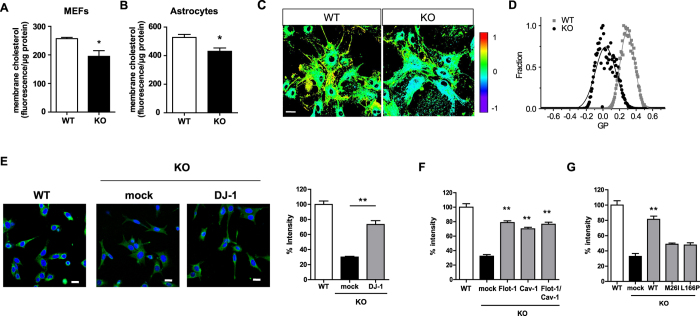
Alterations in the total cholesterol level, membrane fluidity, and lipid rafts-dependent endocytosis. The total cholesterol level was measured in WT and DJ-1 KO MEF cells (**A**), WT and DJ-1 KO primary astrocytes (**B**), as described in ‘Methods’. Data are representative of three independent experiments. *p < 0.05 against WT by an unpaired t-test. (**C,D**) WT and DJ-1 KO primary astrocytes were stained with 0.5 μM C-laurdan, and then observed by two-photon microscopy. Images were processed to obtain GP values as described in ‘Methods’. Scale bar indicates 20 μm. (**E**) DJ-1 KO MEF cells were transfected with DJ-1-myc, (**F**) DJ-1 KO MEF cells were transfected with flot-1 and/or cav-1, respectively, (**G**) DJ-1 KO MEF cells were transfected with WT DJ-1, or DJ-1 mutants (M26I and L166P), respectively, and incubated with 50 nM BOIPY FL C_5_-lactosylceramide (LacCer, green) for 10 minutes. The cells were fixed and observed using confocal microscopy. Blue indicates DAPI. The intensity of LacCer was analyzed by the ImageJ program. Scale bar indicates 20 μm. Values obtained are from three (**E,F**) or four independent experiments (**G**). **p < 0.01 against mock transfectant in DJ-1 KO cells by one-way ANOVA.

Flot-1 and cav-1 have been shown to regulate cholesterol transport^34^,^35^. Accordingly, we measured the total cellular cholesterol level in DJ-1 KO MEF cells. As shown in Fig. 5A, the total cholesterol level in DJ-1 KO MEF cells was lower than that in WT MEF cells, suggesting that the decreased amount of flot-1 and cav-1 caused by DJ-1 deficiency altered the total cholesterol level. We also observed a similar alteration in the total cholesterol level in DJ-1 KO astrocytes (Fig. 5B). In addition, the cholesterol level influences membrane fluidity^36^. Upon measurement of membrane fluidity using C-laurdan^37^, the GP value in DJ-1 KO astrocytes was much lower than that in WT astrocytes, indicating an increase in membrane fluidity (Fig. 5C,D). This suggests that DJ-1 deficiency altered the total cellular cholesterol level, causing an alteration in membrane fluidity.

### DJ-1 regulates lipid raft-dependent endocytosis via flot-1 and cav-1

Alteration in membrane fluidity affects endocytosis^36^. In agreement with previous study showing that DJ-1 regulates lipid rafts-dependent endocytosis in astrocytes^24^, lactosylceramide (LacCer), a marker for lipid rafts-dependent endocytosis^38^ showed less endocytosis in DJ-1 KO MEF cells than in WT MEF cells. In addition, lipid rafts-dependent endocytosis was rescued by overexpression of DJ-1 (Fig. 5E). To explore whether alterations in flot-1 and cav-1 expression caused by DJ-1 contribute to the decrease in lipid rafts-dependent endocytosis shown in DJ-1 KO MEF cells, we overexpressed flot-1 and cav-1 in DJ-1 KO MEF cells. Overexpression of flot-1 and/or cav-1 rescued the decreased lipid rafts-dependent endocytosis shown in DJ-1 KO MEF cells (Fig. 5F). Moreover, overexpression of DJ-1 mutants (M26I and L166P) did not rescue lipid rafts-dependent endocytosis (Fig. 5G), which correlates well with the data shown in Figs 2B and 4C. These data suggest that the decrease in lipid rafts-dependent endocytosis caused by DJ-1 deficiency was mediated by alterations in flot-1 and cav-1, and lipid rafts-dependent endocytosis may be reduced in familial PD patients with mutations in DJ-1.

### DJ-1 regulates glutamate uptake into astrocytes through regulating EAAT2 expression

**Figure 6:**
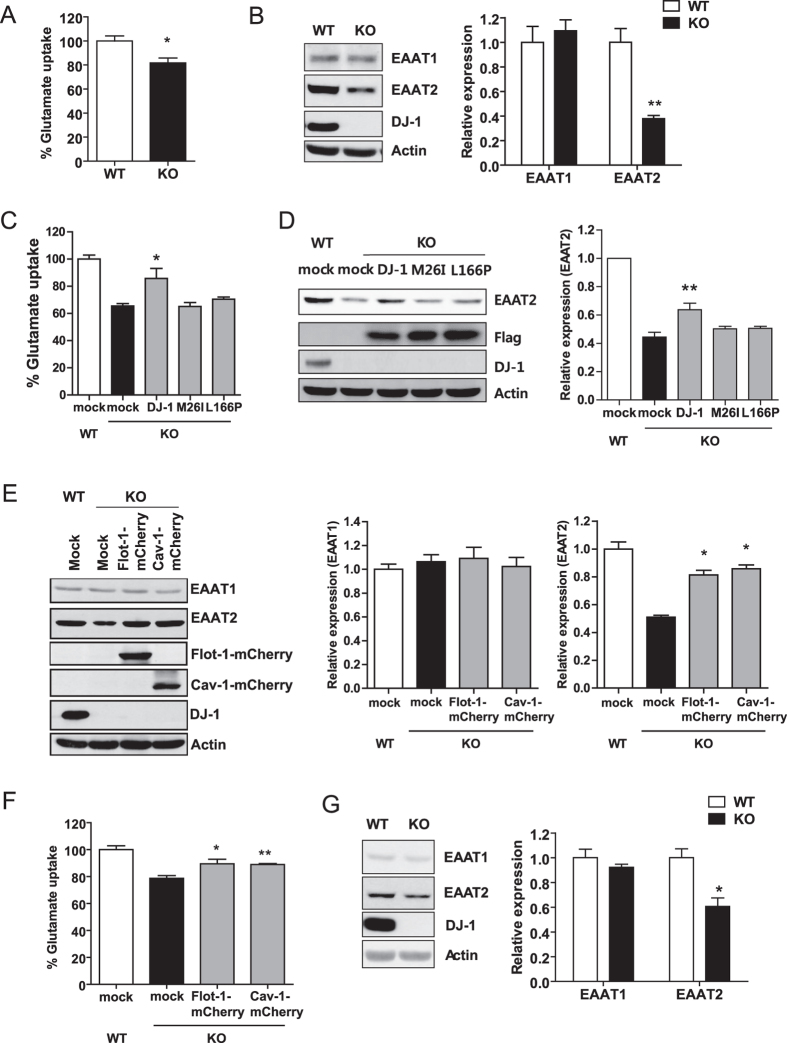
DJ-1 regulates glutamate uptake into astrocytes through the regulation of EAAT2 expression. (**A**) Glutamate uptake was measured as described in ‘Methods’. Data are representative of three independent experiments (n = 3). (**B**) WT and DJ-1 KO primary astrocytes were lysed and the lysates were analyzed for EAAT1 and EAAT2 by Western blotting. Values obtained are from three independent experiments. *p < 0.05, **p < 0.01 against WT by an unpaired t-test. DJ-1 KO primary astrocytes were transfected with flag-tagged WT DJ-1 and DJ-1 mutants (M26I and L166P), and (**C**) glutamate uptake was measured. (**D**) The cells were then lysed and the lysates were analyzed for EAAT2 by Western blotting. DJ-1 KO primary astrocytes were transfected with flot-1-mCherry and cav-1-mCherry. (**E**) The cells were then lysed and the lysates were analyzed for EAAT1 and EAAT2 by Western blotting, and (**F**) glutamate uptake was measured. Values obtained are from three independent experiments. *p < 0.05, **p < 0.01 against mock transfectant in DJ-1 KO cells by one-way ANOVA. (**G**) Whole brain lysates of WT and DJ-1 KO mice were analyzed for EAAT1 and EAAT2 by Western blotting. *p < 0.05 against WT by an unpaired t-test (n = 3).

DJ-1 deficiency altered membrane fluidity, which may interfere with a variety of lipid rafts-associated signaling pathways. It has been reported that EAAT2, a major glutamate transporter expressed in astrocytes, associates with lipid rafts and this association is important for its function^39^,^40^. Accordingly, we explored whether glutamate uptake into astrocytes is altered by DJ-1 deficiency. As shown in Fig. 6A, glutamate uptake was impaired in DJ-1 KO astrocytes compared with WT astrocytes. In addition, the expression level of the major glutamate transporters, EAAT1 and EAAT2^41^ was assessed. Interestingly, the expression of EAAT2 was decreased in DJ-1 KO astrocytes compared with WT astrocytes, however, the expression of EAAT1 was not (Fig. 6B). The expression of EAAT2 was decreased in both the soluble and insoluble fractions of DJ-1 KO astrocytes (Supplementary Fig. 1). In addition, overexpression of WT DJ-1 in DJ-1 KO astrocytes rescued the decrease in glutamate uptake and EAAT2 expression, but overexpression of familial mutants (M26I and L166P) did not (Fig. 6C,D). To investigate whether an alteration in flot-1 and cav-1 contributed to a decrease in EAAT2 expression in astrocytes, flot-1-mCherry and cav-1-mCherry were overexpressed in DJ-1 KO astrocytes. Overexpression of flot-1-mCherry and cav-1-mCherry rescued the decreased expression of EAAT2, however, did not affect EAAT1 expression (Fig. 6E). In addition, overexpression of flot-1-mCherry and cav-1-mCherry rescued the decrease in glutamate uptake into DJ-1 KO astrocytes (Fig. 6F), suggesting that alterations in flot-1 and cav-1 expression by DJ-1 deficiency contribute to the impairment of glutamate uptake into DJ-1 KO astrocytes. Finally, we observed decreased EAAT2 in brain lysates from DJ-1 KO mice, compared with those from WT mice (Fig. 6G). These data suggest that DJ-1 regulates glutamate uptake in astrocytes via flot-1 and cav-1.

## Discussion

Increasing evidence indicates that alterations in lipid rafts are associated with many neurodegenerative diseases including Alzheimer’s disease (AD), Huntington’s disease (HD), and amyotrophic lateral sclerosis (ALS) as well as PD^27^,^42^,^43^. Altered lipid composition in cortical lipid rafts occurs in AD^44^ and PD^25^, and altered protein composition in lipid rafts occurs in murine models of AD^45^ and ALS^46^. Accordingly, it was proposed that altered lipid rafts may be a common pathology that causes the neurodegeneration shown in neurodegenerative diseases^27^,^42^.

In the present study, we demonstrate that DJ-1 regulates flot-1 and cav-1 expression, which are the main protein components of lipid rafts. Flot-1 is a member of the flotillin family, composed of flot-1 and -2, and is considered to be a scaffolding protein of lipid rafts, generally being used as a marker protein of lipid rafts^47^. Although their functions are still controversial, they are known to be involved in the trafficking and targeted delivery of membrane and membrane proteins to very specific sites in a cell type-specific manner^48^. Cav-1 is a member of the caveolin family, composed of cav-1, -2, and -3, and is also considered to be a scaffolding protein of caveolae, a subset of lipid rafts^49^, functioning in membrane-initiated intracellular signaling via the clustering and segregation of proteins, and the trafficking of proteins and lipids^50^,^51^, which is similar with flot-1. We observed that flot-1 and cav-1 are specifically downregulated in DJ-1 KO MEF cells and primary astrocytes, which was also observed in DJ-1 KO brain lysates.

Regarding the mechanism of regulation of flot-1 and cav-1 by DJ-1, we demonstrate that DJ-1 regulated flot-1 stability by direct interaction, however, decreased cav-1 expression may not be a direct effect of DJ-1, but rather as a result of decreased flot-1 expression. Interestingly, flot-1 which binds to DJ-1 has slightly different mobility compared with that in total lysates, implying that DJ-1 may interact with post-translationally modified flot-1, such as by phosphorylation. Although we conclude that decreased cav-1 expression may not be a direct effect of DJ-1, we cannot exclude other mechanisms completely. It has been reported that DJ-1 regulates SOCS1 expression through the regulation of miR-155 levels^52^. MicroRNAs (miRNAs) are endogenous noncoding small RNAs that contribute to the regulation of their target gene, resulting in mRNA degradation or translational inhibition^53^. According to a prediction program (PITA (http://genie.weizmann.ac.il/pubs/mir07/mir07_prediction.html)), we found that flot-1 and cav-1 expression can be regulated by common miRNAs, miR-124, and -138. It has also been reported that miR-124, and -138 regulate flot-1 and cav-1 expression^54^,^55^, indicating that DJ-1 may regulate flot-1 and cav-1 via miRNAs. Accordingly, as another possible mechanism of the regulation of flot-1 and cav-1 expression by DJ-1, we explored the level of miR-124, and -138 between WT and DJ-1 KO MEF cells. However, we did not observe any difference in miR-124, and -138 levels between the two cell lines (Supplementary Fig. 2), suggesting that the regulation of flot-1 and cav-1 expression by DJ-1 is not mediated by miRNAs. Further study is needed to clarify the molecular mechanism by which DJ-1 regulates cav-1 expression.

In addition, we demonstrate that the total cellular cholesterol level and membrane fluidity were altered by DJ-1 deficiency, causing defects in lipid rafts-dependent endocytosis, which is rescued by flot-1 and cav-1 overexpression. This suggests that dysregulation of lipid rafts-dependent endocytosis by DJ-1 deficiency was derived from the abnormal regulation of flot-1 and cav-1 expression, and these results can explain the molecular mechanism underlying our previous findings that DJ-1 regulates lipid rafts-dependent endocytosis in astrocytes^24^. Interestingly, we observed that familial mutants of DJ-1 did not rescue flot-1 or cav-1 expression in DJ-1 KO MEF cells and astrocytes, and less interacted with flot-1. In addition, the mutants did not rescue decreased lipid rafts-dependent endocytosis in DJ-1 KO MEF cells, suggesting that it is highly possible that the dysregulation of flot-1 and cav-1 expression contributes to the pathogenesis of PD. Flotillins have been identified as regeneration molecules upregulated in the regenerating axons of retinal ganglion cells after lesions of the optic nerve^56^. The downregulation of flotillins has been shown to trigger a clear reduction in the number of regenerating axons in zebrafish, indicating that flotillins are indeed necessary for axon regeneration^57^. Jacobowitz and Kallarakal observed that the expression of flot-1 was upregulated in the substantia nigra of patients with PD, and proposed that this may be a possible attempt of the nigrostriatal system to innervate and sprout new fibers in the striatum^58^. This suggests that the downregulation of flot-1 may cause neurodegeneration, and partially supports our speculation of the association between flot-1 and PD.

In our previous study, we demonstrated that DJ-1 regulates inflammation through the regulation of lipid rafts-dependent endocytosis in astrocytes^24^. Considering that DJ-1 regulates flot-1 and cav-1, the main protein components of lipid rafts, it can be speculated that DJ-1 deficiency may cause many more defects associated with lipid rafts. One of the major functions of astrocytes is the regulation of extracellular glutamate levels through glutamate uptake into astrocytes by glutamate transporters^59^. Glutamate uptake by EAATs plays an important role in reducing the glutamate concentration in the extracellular space. Astrocytes express EAAT1 and EAAT2 as their main glutamate transporters^60^. It has been reported that a large proportion of the total EAAT2 and a minor proportion of the total EAAT1 are associated with lipid rafts, and the function of glutamate transporters is associated with lipid rafts^39^, indicating that DJ-1 may regulate glutamate clearance through glutamate transporters associated with lipid rafts. Supporting our speculation, decreased glutamate uptake was observed in DJ-1 KO primary astrocytes, which may cause abnormal clearance of extracellular glutamate under stress conditions such as stroke, further followed by excitotoxicity. Previous findings showing that loss of DJ-1 increases the sensitivity to excitotoxicity in an *in vivo* stroke model^61^ correlate well with our data. In addition, EAAT2, but not EAAT1, was specifically downregulated in DJ-1 KO primary astrocytes, and overexpression of cav-1 and flot-1 rescued the decreased glutamate uptake and decreased expression of EAAT2 by DJ-1 deficiency. Although EAAT2 expression was highly decreased in DJ-1 KO primary astrocytes, impaired glutamate uptake was less severe, which may be explained by the intact function of EAAT1. In addition, partial rescues by overexpression of cav-1 and flot-1 can be explained by the transfection efficiency in primary astrocytes. This suggests that EAAT2 may be mistrafficked by an alteration in lipid rafts, resulting in degradation, which is in agreement with a previous report regarding the role of flot-1 in Erb2 expression^62^. Accordingly, it suggests that functional DJ-1 deficiency, including mutations found in PD patients, may disrupt a more broad variety of lipid rafts-associated membrane signaling, which cause the neurodegeneration seen in PD. Previous reports indicating a decreased EAAT2 expression in animal models of PD, including the 6-hydroxydopamine lesioned PD model^63^ and the acute 1-methyle-4-phenyl-1,2,3,6-tetrahydropyridine treated model^64^, also partially support our data.

In conclusion, we have demonstrated that DJ-1 deficiency induced a decrease in flot-1 and cav-1 expression. DJ-1 regulated flot-1 stability by direct interaction, however, decreased cav-1 expression may be due to decreased flot-1 expression, rather than DJ-1 directly. Dysregulation of flot-1 and cav-1 by DJ-1 deficiency caused an alteration in the total cellular cholesterol level and membrane fluidity, and further alteration in lipid rafts-dependent endocytosis. Moreover, DJ-1 deficiency impairs glutamate uptake into astrocytes by altering EAAT2 expression. The present study will be helpful in the understanding of the role of DJ-1 in the pathogenesis of PD, and the modulation of lipid rafts by regulating flot-1 or cav-1 may be a novel therapeutic target for PD.

## Methods

### Reagents and antibodies

Antibodies against caveolin-1, caveolin-2, flotillin-1 and flotillin-2 for Western blotting were purchased from BD Bioscience (Franklin Lakes, NJ) and antibodies against caveolin-1 and flotillin-1 for immunocytochemistry were purchased from Abcam (Cambridge, MA). Antibodies against the transferrin receptor (CD71), β-actin, EAAT1, EAAT2 and DJ-1 were purchased from Santa Cruz Biotechnology (CA, USA). BOIPY^®^ FL C_5_-Lactosylceramide was purchased from Molecular Probes (Leiden, the Netherlands). L-[3,4-^3^H] glutamic acid was purchased from PerkinElmer (Boston, MA, USA). MG132 was purchased from Sigma (St Louis, MO). Cycloheximide was purchased from Calbiochem (Danvers, MA).

### Constructs

p3XFlag-WT, M26I, E64D, and L166P DJ-1 constructs were prepared previously^24^. pFlot-1-mCherry was kindly provided by Dr. Verena Niggli, Department of Pathology, University of Bern, Switzerland. pCav-1-mCherry and pCDNA3.1-DJ-1-myc. His were generated using PCR. All constructs were verified by DNA sequencing and were prepared by an Endo-Free plasmid Maxi prep Kit (Qiagen, Valencia, CA) to remove endotoxin contamination.

### Cell culture and transfection

DJ-1 KO mice were kindly provided by Dr. UJ Kang, Department of Neurology, University of Chicago. DJ-1 WT and KO mouse embryo fibroblasts (MEFs) were kindly provided by Dr. Tak Mak, Department of Medical Biophysics, University of Toronto, Canada. Mouse primary astrocytes, isolated from cerebral cortical tissues of 1 day postnatal mice, were cultured as previously described^24^. MEF cells were grown in Dulbecco’s modified Eagle’s medium (DMEM) supplemented with 10% fetal bovine serum (FBS) in a humidified atmosphere under 5% CO_2_ at 37 °C. MEF cells and primary astrocytes were transfected using Lipofectamine 2000 (Invitrogen, Carlsbad, CA) according to the manufacturer’s instructions. Twenty four hours post-transfection, the cells were used for further experiments. All animal procedures used in the present study were carried out in accordance with the guidelines approved by the Ajou University School of Medicine Ethics Review Committee for Animal Experiments, and all experimental protocols were approved by the Ajou University School of Medicine Review Committee for Animal Experiments.

### Western blotting and co-immunoprecipitation

Cell extracts were prepared in RIPA buffer (50 mM Tris-HCl, pH 7.4, 1% Nonidet P-40, 0.25% sodium deoxycholate, 150 mM NaCl, and 1 mM Na_3_VO_4_) containing protease inhibitors (2 mM phenylmethylsulfonyl fluoride, 100 μg/ml leupeptin, 10 μg/ml pepstatin, 1 μg/ml aprotinin, and 2 mM EDTA) and phosphatase inhibitor cocktail (GenDEPOT, Baker, TX). After the cell extracts were incubated for 20 minutes at 4 °C, the lysates were centrifuged at 12,000 rpm for 20 minutes at 4 °C, and the supernatants were collected for Western blotting. The protein concentration was measured using a BCA protein assay kit (Bio-rad, USA). The lysates were separated by sodium dodecyl sulfate-polyacrylamide gel electrophoresis (SDS-PAGE) and transferred to nitrocellulose membranes. Membranes were incubated with 5% skimmed milk for 1 hour at room temperature, and then incubated with primary antibodies overnight at 4 °C. After washing extensively, membranes were then incubated with the horseradish peroxidase (HRP)-conjugated secondary antibody (Jackson Immunoresearch, West Grove, PA). The signal was detected using WESTSAVE^TM^ (Ab FRONTIER, Seoul, Korea), and the enhanced chemiluminescence (ECL) system.

For co-immunoprecipitation, 1 mg each supernatant was incubated with 1 μg primary antibody overnight at 4 °C, which was subsequently adsorbed onto protein G-agarose (Millipore, Temecula, CA). After extensive washing with ice-cold RIPA buffer, the samples were prepared in 2X SDS sample buffer for 10 minutes and loaded onto gels for SDS-PAGE followed by Western blotting.

### Isolation of the lipid rafts fraction

Cells were washed twice with ice-cold phosphate buffered saline (PBS) and lysed in ice-cold 1% Triton X-100 buffer containing protease inhibitor cocktail (GenDEPOT, Barker, TX). Following incubation of the cells for 10 minutes at 4 °C, the lysates were centrifuged at 12,000 rpm for 20 minutes at 4 °C. Supernatants were used as the soluble fraction. The pellets were washed with ice-cold PBS buffer, solubilized in 1X SDS sample buffer and used as the insoluble fraction. The protein concentration for the soluble fraction was measured using a BCA protein assay kit and then the loading volume for the insoluble fraction was determined. These individual fractions were analyzed by SDS-PAGE and Western blotting.

### Preparation of brain lysates

Brain tissues from WT and DJ-1 KO mice were isolated at the age of 10 weeks. Whole brain tissues were lysed by homogenization in 1 ml RIPA buffer containing protease inhibitor and phosphatase inhibitor cocktail. Following incubation for 20 minutes at 4 °C, the tissues were sonicated, and the lysates were centrifuged at 12,000 rpm for 20 minutes at 4 °C. The supernatants were collected for Western blotting. The protein concentration was measured using a BCA protein assay kit.

### Immunocytochemistry

Cells grown on a glass coverslip were fixed with 4% paraformaldehyde for 10 minutes at room temperature. The fixed cells were then washed with PBS and permeabilized with PBS containing 0.1% Triton X-100 for 10 minutes at room temperature. After washing several times with PBS, the cells were blocked with PBS containing 5% BSA for 1 hour at room temperature. The cells were then incubated with primary antibody for 1 hour at room temperature, followed by incubation with Alexa 488- or Alexa 568- conjugated secondary antibodies (Jackson Immunoresearch, West Grove, PA) for 2 hours and Hoechst for 10 minutes for nuclear staining. The coverslips were then mounted and observed under a confocal microscope (Zeiss, Germany).

### *In situ* proximity ligations assay (PLA)

The PLA was performed using the DuoLink PLA kit (Sigma-Aldrich, St. Louis, MO) according to the manufacturer’s instructions. Briefly, cells were fixed with 4% paraformaldehyde and permeabilized with 0.1% Triton X-100. Following treatment with DuoLink blocking buffer, cells were incubated with diluted primary antibodies against DJ-1 and flot-1. After washing, cells were incubated with species-specific PLA probes and two additional oligonucleotides under hybridization conditions. Hybridization occurs when PLA probes are in close proximity, which can be subsequently ligated to form a closed circle. A rolling-circle amplification step follows with polymerase to generate a concatemeric product, which can be visualized with fluorophore-labeled oligonucleotides after hybridization. The slides were stained with DAPI and analyzed using confocal microscopy (Zeiss, Germany).

### Quantitative real-time PCR

Total RNA was isolated from cells, and reverse-transcription was performed to synthesize cDNA using avian myeloblastosis virus (AMV) RT (Promega, Madison, WI). cDNA samples were analyzed by 2 × KAPA SYBR Fast Master Mix (Kapa Biosystems, Cape Town, South Africa) on Rotor-Gene cyclers (Corbett Research, Sydney, Australia) with specific primers. The primer sequences for PCR were as follows: forward, 5′-CGTAGACTCCGAGGGACATC-3′, and reverse, 5′-TCCCTTCTGGTTCTGCAATC-3′, for mouse cav-1; forward, 5′-AGAAGCCTTCCAGATGTACC-3′, and reverse, 5′-ATGTCCAGTACTTCCCCAGT-3′, for mouse flot-1; forward, 5′-TGTTACCAACTGGGACGACA-3′, and reverse, 5′-GGGGTGTTGAAGGTCTCAAA-3′ for actin as an internal control. The values of mRNA were calculated using the delta Ct method and expressed as a change relative to the expression of actin mRNA as an internal control.

### Measurement of the total cellular cholesterol

The membrane cholesterol was measured using the Amplex Red cholesterol assay kit (Molecular probe, Eugene, OR). MEF cells and mouse primary astrocytes were lysed in 1x reaction buffer (0.1 M potassium phosphate, pH 7.4, 50 mM NaCl, 5 mM cholic acid, 0.1% Triton X-100) for 30 minutes at 4 °C and sonicated. The lysates were incubated with a working solution of 300 μM Amplex Red reagent containing 2 U/ml HRP, 2 U/ml cholesterol oxidase, and 0.2 U/ml cholesterol esterase in 1x reaction buffer for 10 minutes at room temperature. Fluorescence was measured using a microplate reader (λ_ex_ = 530 nm, λ_em_ = 590 nm). The protein concentration was measured using a BCA protein assay kit. The membrane cholesterol levels are expressed as specific fluorescence/mg protein.

### Measurement of membrane fluidity using C-laurdan

Primary astrocytes from WT and DJ-1 KO mice cultured on glass-bottomed culture dishes were incubated with culture media containing 0.5 μM C-laurdan (SFP Co., Ltd, Chungbuk, Korea) for 20 minutes at 37 °C. The membrane fluidity observed using two-photon fluorescence images was obtained with spectral confocal multiphoton microscopes (Leica TCS SP2, Wetzlar, Germany). The intensity images of C-laurdan were recorded with the emission in the range of 400–460 nm and 470–530 nm with two channels of PMT. Generalized polarization (GP) values for each pixel were obtained using the GP analysis program, an ImageJ plugin (http://imagej.nih.gov/ij/), and were processed as described previously^37^. GP values range from −1, corresponding to the highest fluidity, to +1 for the lowest fluidity.

### *In vitro* lipid rafts-dependent endocytosis assay

WT and DJ-1 KO MEF cells were transfected with WT DJ-1, mCherry-tagged flot-1 and cav-1, and Flag-tagged WT DJ-1 and DJ-1 mutants (M26I, and L166P). The cells were incubated with 50 nM BOIPY FL C_5_-lactosylceramide for 10 minutes, fixed, and incubated with Hoechst for nuclear staining. Subsequently, they were mounted and observed under a confocal microscope (Zeiss, Germany). The intensity of LacCer was analyzed by the ImageJ (http://imagej.nih.gov/ij/) program.

### Glutamate uptake assay

Primary astrocytes from WT and DJ-1 KO mice were cultured in 24-well plates to measure the uptake of L-[3,4-^3^H] glutamic acid. Cells were incubated with media containing 0.4 μCi L-[3,4-^3^H] glutamic acid for 10 minutes at room temperature. The media were then removed, and the cells were washed twice with ice-cold PBS in order to terminate glutamic acid uptake. Cells were lysed with 0.5 ml 1 N NaOH, and the accumulated radiolabeled glutamic acid was measured using liquid scintillation counting. The concentration of total cell protein was used as a reference, which was determined using a BCA protein assay kit.

### Statistical analysis

All values are expressed as the mean ± SEM. Statistical significance was evaluated using the Graphpad software (San Diego, CA).

## Additional Information

**How to cite this article**: Kim, J.-M. *et al*. DJ-1 deficiency impairs glutamate uptake into astrocytes via the regulation of flotillin-1 and caveolin-1 expression. *Sci. Rep.* **6**, 28823; doi: 10.1038/srep28823 (2016).

## Supplementary Material

Supplementary Information

## References

[b1] Kalia L. V. & Lang A. E. Parkinson’s disease. Lancet, 10.1016/S0140-6736(14)61393-3 (2015).

[b2] Lees A. J., Hardy J. & Revesz T. Parkinson’s disease. Lancet 373, 2055–2066, 10.1016/s0140-6736(09)60492-x (2009). 19524782

[b3] Nuytemans K., Theuns J., Cruts M. & Van Broeckhoven C. Genetic etiology of Parkinson disease associated with mutations in the SNCA, PARK2, PINK1, PARK7, and LRRK2 genes: a mutation update. Hum Mutat 31, 763–780, 10.1002/humu.21277 (2010). 20506312 PMC3056147

[b4] Saiki S., Sato S. & Hattori N. Molecular pathogenesis of Parkinson’s disease: update. J Neurol Neurosurg Psychiatry 83, 430–436, 10.1136/jnnp-2011-301205 (2012). 22138181

[b5] Bonifati V. . Mutations in the DJ-1 gene associated with autosomal recessive early-onset parkinsonism. Science 299, 256–259, 10.1126/science.1077209 (2003). 12446870

[b6] da Costa C. A. DJ-1: a newcomer in Parkinson’s disease pathology. Curr Mol Med 7, 650–657 (2007). 18045143 10.2174/156652407782564426

[b7] Lee S. J. . Crystal structures of human DJ-1 and *Escherichia coli* Hsp31, which share an evolutionarily conserved domain. J Biol Chem 278, 44552–44559, 10.1074/jbc.M304517200 (2003). 12939276

[b8] Kim J. H. . DJ-1 facilitates the interaction between STAT1 and its phosphatase, SHP-1, in brain microglia and astrocytes: A novel anti-inflammatory function of DJ-1. Neurobiol Dis 60, 1–10, 10.1016/j.nbd.2013.08.007 (2013). 23969237

[b9] Im J. Y., Lee K. W., Woo J. M., Junn E. & Mouradian M. M. DJ-1 induces thioredoxin 1 expression through the Nrf2 pathway. Hum Mol Genet 21, 3013–3024, 10.1093/hmg/dds131 (2012). 22492997 PMC3373246

[b10] Takahashi K. . DJ-1 positively regulates the androgen receptor by impairing the binding of PIASx alpha to the receptor. J Biol Chem 276, 37556–37563, 10.1074/jbc.M101730200 (2001). 11477070

[b11] Mitsumoto A. & Nakagawa Y. DJ-1 is an indicator for endogenous reactive oxygen species elicited by endotoxin. Free Radic Res 35, 885–893 (2001). 11811539 10.1080/10715760100301381

[b12] Taira T. . DJ-1 has a role in antioxidative stress to prevent cell death. EMBO Rep 5, 213–218, 10.1038/sj.embor.7400074 (2004). 14749723 PMC1298985

[b13] Usami Y. . DJ-1 associates with synaptic membranes. Neurobiol Dis 43, 651–662, 10.1016/j.nbd.2011.05.014 (2011). 21645620

[b14] Zhang L. . Mitochondrial localization of the Parkinson’s disease related protein DJ-1: implications for pathogenesis. Hum Mol Genet 14, 2063–2073, 10.1093/hmg/ddi211 (2005). 15944198

[b15] Ariga H. . Neuroprotective Function of DJ-1 in Parkinson’s Disease. Oxid Med Cell Longev 2013, 683920, 10.1155/2013/683920 (2013). 23766857 PMC3671546

[b16] Koyano F. . Ubiquitin is phosphorylated by PINK1 to activate parkin. Nature 510, 162–166, 10.1038/nature13392 (2014). 24784582

[b17] Lin X. . Leucine-rich repeat kinase 2 regulates the progression of neuropathology induced by Parkinson’s-disease-related mutant alpha-synuclein. Neuron 64, 807–827, 10.1016/j.neuron.2009.11.006 (2009). 20064389 PMC2807409

[b18] Smith W. W. . Leucine-rich repeat kinase 2 (LRRK2) interacts with parkin, and mutant LRRK2 induces neuronal degeneration. Proc Natl Acad Sci USA 102, 18676–18681, 10.1073/pnas.0508052102 (2005). 16352719 PMC1317945

[b19] Simons K. & Toomre D. Lipid rafts and signal transduction. Nat Rev Mol Cell Biol 1, 31–39, 10.1038/35036052 (2000). 11413487

[b20] Fortin D. L. . Lipid rafts mediate the synaptic localization of alpha-synuclein. J Neurosci 24, 6715–6723, 10.1523/jneurosci.1594-04.2004 (2004). 15282274 PMC6729723

[b21] Fallon L. . Parkin and CASK/LIN-2 associate via a PDZ-mediated interaction and are co-localized in lipid rafts and postsynaptic densities in brain. J Biol Chem 277, 486–491, 10.1074/jbc.M109806200 (2002). 11679592

[b22] Silvestri L. . Mitochondrial import and enzymatic activity of PINK1 mutants associated to recessive parkinsonism. Hum Mol Genet 14, 3477–3492, 10.1093/hmg/ddi377 (2005). 16207731

[b23] Hatano T. . Leucine-rich repeat kinase 2 associates with lipid rafts. Hum Mol Genet 16, 678–690, 10.1093/hmg/ddm013 (2007). 17341485

[b24] Kim K. S. . DJ-1 associates with lipid rafts by palmitoylation and regulates lipid rafts-dependent endocytosis in astrocytes. Hum Mol Genet 22, 4805–4817, 10.1093/hmg/ddt332 (2013). 23847046

[b25] Fabelo N. . Severe alterations in lipid composition of frontal cortex lipid rafts from Parkinson’s disease and incidental Parkinson’s disease. Mol Med 17, 1107–1118, 10.2119/molmed.2011.00119 (2011). 21717034 PMC3188884

[b26] Kubo S., Hatano T. & Hattori N. Lipid rafts involvement in the pathogenesis of Parkinson’s disease. Front Biosci (Landmark Ed.) 20, 263–279 (2015). 25553450 10.2741/4308

[b27] Sonnino S. . Lipid rafts in neurodegeneration and neuroprotection. Mol Neurobiol 50, 130–148, 10.1007/s12035-013-8614-4 (2014). 24362851

[b28] El-Sayed A. & Harashima H. Endocytosis of gene delivery vectors: from clathrin-dependent to lipid raft-mediated endocytosis. Mol Ther 21, 1118–1130, 10.1038/mt.2013.54 (2013). 23587924 PMC3677298

[b29] Gong Q., Huntsman C. & Ma D. Clathrin-independent internalization and recycling. J Cell Mol Med 12, 126–144, 10.1111/j.1582-4934.2007.00148.x (2008). 18039352 PMC3823476

[b30] Zlatkine P., Mehul B. & Magee A. I. Retargeting of cytosolic proteins to the plasma membrane by the Lck protein tyrosine kinase dual acylation motif. J Cell Sci 110 (Pt 5), 673–679 (1997). 9092949 10.1242/jcs.110.5.673

[b31] Chen S. F. . Caveolin-1 facilitates cyclooxygenase-2 protein degradation. J Cell Biochem 109, 356–362, 10.1002/jcb.22407 (2010). 19960513

[b32] Solis G. P. . Reggie/flotillin proteins are organized into stable tetramers in membrane microdomains. Biochem J 403, 313–322, 10.1042/BJ20061686 (2007). 17206938 PMC1874235

[b33] Vassilieva E. V., Ivanov A. I. & Nusrat A. Flotillin-1 stabilizes caveolin-1 in intestinal epithelial cells. Biochem Biophys Res Commun 379, 460–465, 10.1016/j.bbrc.2008.12.118 (2009). 19121286 PMC2867594

[b34] Ge L. . Flotillins play an essential role in Niemann-Pick C1-like 1-mediated cholesterol uptake. Proc Natl Acad Sci USA 108, 551–556, 10.1073/pnas.1014434108 (2011). 21187433 PMC3021008

[b35] Fu Y. . Expression of caveolin-1 enhances cholesterol efflux in hepatic cells. J Biol Chem 279, 14140–14146, 10.1074/jbc.M311061200 (2004). 14729661

[b36] Spector A. A. & Yorek M. A. Membrane lipid composition and cellular function. J Lipid Res 26, 1015–1035 (1985). 3906008

[b37] Kim H. M. . A two-photon fluorescent probe for lipid raft imaging: C-laurdan. Chembiochem 8, 553–559, 10.1002/cbic.200700003 (2007). 17300111

[b38] Marks D. L., Singh R. D., Choudhury A., Wheatley C. L. & Pagano R. E. Use of fluorescent sphingolipid analogs to study lipid transport along the endocytic pathway. Methods 36, 186–195, 10.1016/j.ymeth.2004.12.001 (2005). 15905102

[b39] Butchbach M. E., Tian G., Guo H. & Lin C. L. Association of excitatory amino acid transporters, especially EAAT2, with cholesterol-rich lipid raft microdomains: importance for excitatory amino acid transporter localization and function. J Biol Chem 279, 34388–34396, 10.1074/jbc.M403938200 (2004). 15187084

[b40] Tian G., Kong Q., Lai L., Ray-Chaudhury A. & Lin C. L. Increased expression of cholesterol 24S-hydroxylase results in disruption of glial glutamate transporter EAAT2 association with lipid rafts: a potential role in Alzheimer’s disease. J Neurochem 113, 978–989, 10.1111/j.1471-4159.2010.06661.x (2010). 20193040 PMC3010752

[b41] Lehre K. P., Levy L. M., Ottersen O. P., Storm-Mathisen J. & Danbolt N. C. Differential expression of two glial glutamate transporters in the rat brain: quantitative and immunocytochemical observations. J Neurosci 15, 1835–1853 (1995). 7891138 10.1523/JNEUROSCI.15-03-01835.1995 PMC6578153

[b42] Schengrund C. L. Lipid rafts: keys to neurodegeneration. Brain Res Bull 82, 7–17, 10.1016/j.brainresbull.2010.02.013 (2010). 20206240

[b43] Marin R., Rojo J. A., Fabelo N., Fernandez C. E. & Diaz M. Lipid raft disarrangement as a result of neuropathological progresses: a novel strategy for early diagnosis? Neuroscience 245, 26–39, 10.1016/j.neuroscience.2013.04.025 (2013). 23618758

[b44] Fabelo N. . Altered lipid composition in cortical lipid rafts occurs at early stages of sporadic Alzheimer’s disease and facilitates APP/BACE1 interactions. Neurobiol Aging 35, 1801–1812, 10.1016/j.neurobiolaging.2014.02.005 (2014). 24613671

[b45] Chadwick W., Brenneman R., Martin B. & Maudsley S. Complex and multidimensional lipid raft alterations in a murine model of Alzheimer’s disease. Int J Alzheimers Dis 2010, 604792, 10.4061/2010/604792 (2010). 21151659 PMC2997345

[b46] Zhai J. . Proteomic characterization of lipid raft proteins in amyotrophic lateral sclerosis mouse spinal cord. FEBS J 276, 3308–3323, 10.1111/j.1742-4658.2009.07057.x (2009). 19438725 PMC2754564

[b47] Zhao F., Zhang J., Liu Y. S., Li L. & He Y. L. Research advances on flotillins. Virol J 8, 479, 10.1186/1743-422X-8-479 (2011). 22023811 PMC3215287

[b48] Stuermer C. A. Reggie/flotillin and the targeted delivery of cargo. J Neurochem 116, 708–713, 10.1111/j.1471-4159.2010.07007.x (2011). 21214550

[b49] Lajoie P. & Nabi I. R. Lipid rafts, caveolae, and their endocytosis. Int Rev Cell Mol Biol 282, 135–163, 10.1016/S1937-6448(10)82003-9 (2010). 20630468

[b50] Stern C. M. & Mermelstein P. G. Caveolin regulation of neuronal intracellular signaling. Cell Mol Life Sci 67, 3785–3795, 10.1007/s00018-010-0447-y (2010). 20632068 PMC3740547

[b51] Liu P., Rudick M. & Anderson R. G. Multiple functions of caveolin-1. J Biol Chem 277, 41295–41298, 10.1074/jbc.R200020200 (2002). 12189159

[b52] Kim J. H., Jou I. & Joe E. H. Suppression of miR-155 Expression in IFN-gamma-Treated Astrocytes and Microglia by DJ-1: A Possible Mechanism for Maintaining SOCS1 Expression. Exp Neurobiol 23, 148–154, 10.5607/en.2014.23.2.148 (2014). 24963279 PMC4065828

[b53] Kim V. N., Han J. & Siomi M. C. Biogenesis of small RNAs in animals. Nat Rev Mol Cell Biol 10, 126–139, 10.1038/nrm2632 (2009). 19165215

[b54] Gong H. . Downregulation of miR-138 sustains NF-kappaB activation and promotes lipid raft formation in esophageal squamous cell carcinoma. Clin Cancer Res 19, 1083–1093, 10.1158/1078-0432.CCR-12-3169 (2013). 23319823

[b55] Li L. . Microrna-124 targets flotillin-1 to regulate proliferation and migration in breast cancer. Mol Cancer 12, 163, 10.1186/1476-4598-12-163 (2013). 24330780 PMC4029407

[b56] Schulte T., Paschke K. A., Laessing U., Lottspeich F. & Stuermer C. A. Reggie-1 and reggie-2, two cell surface proteins expressed by retinal ganglion cells during axon regeneration. Development 124, 577–587 (1997). 9053333 10.1242/dev.124.2.577

[b57] Munderloh C. . Reggies/flotillins regulate retinal axon regeneration in the zebrafish optic nerve and differentiation of hippocampal and N2a neurons. J Neurosci 29, 6607–6615, 10.1523/jneurosci.0870-09.2009 (2009). 19458231 PMC6665911

[b58] Jacobowitz D. M. & Kallarakal A. T. Flotillin-1 in the substantia nigra of the Parkinson brain and a predominant localization in catecholaminergic nerves in the rat brain. Neurotox Res 6, 245–257 (2004). 15545008 10.1007/BF03033435

[b59] Allen N. J. Astrocyte regulation of synaptic behavior. Annu Rev Cell Dev Biol 30, 439–463, 10.1146/annurev-cellbio-100913-013053 (2014). 25288116

[b60] Rothstein J. D. . Localization of neuronal and glial glutamate transporters. Neuron 13, 713–725 (1994). 7917301 10.1016/0896-6273(94)90038-8

[b61] Aleyasin H. . The Parkinson’s disease gene DJ-1 is also a key regulator of stroke-induced damage. Proc Natl Acad Sci USA 104, 18748–18753, 10.1073/pnas.0709379104 (2007). 18003894 PMC2141848

[b62] Pust S. . Flotillins as regulators of ErbB2 levels in breast cancer. Oncogene 32, 3443–3451, 10.1038/onc.2012.357 (2013). 22869152

[b63] Chung E. K., Chen L. W., Chan Y. S. & Yung K. K. Downregulation of glial glutamate transporters after dopamine denervation in the striatum of 6-hydroxydopamine-lesioned rats. J Comp Neurol 511, 421–437, 10.1002/cne.21852 (2008). 18831527

[b64] Holmer H. K., Keyghobadi M., Moore C. & Meshul C. K. l-dopa-induced reversal in striatal glutamate following partial depletion of nigrostriatal dopamine with 1-methyl-4-phenyl-1,2,3,6-tetrahydropyridine. Neuroscience 136, 333–341, 10.1016/j.neuroscience.2005.08.003 (2005). 16198485

