# Supplementary material for: DJ-1 deficiency impairs glutamate uptake into astrocytes via the regulation of flotillin-1 and caveolin-1 expression
Source: Sci Rep. 2016 Jun 27;6:28823. doi: 10.1038/srep28823 (PMC4922019; doi:10.1038/srep28823)
Supplement: Supplementary Information [file srep28823-s1.pdf]

DJ-1 deficiency impairs glutamate uptake into astrocytes via the regulation of flotillin-1 and caveolin-1 expression

Jin-Mo Kim, Seon-Heui Cha, YuRee Choi, Ilo Jou, Eun-Hye Joe and Sang Myun Park*

Supplementary figure 1, 2

Supplementary figure 1, 2 legend

Methods for supplementary figure 2

Supplementary figure 1.

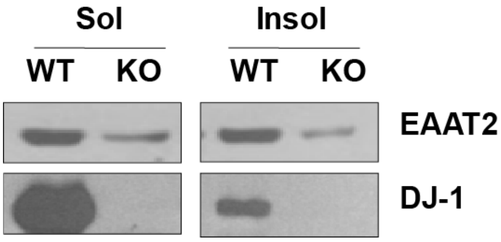

Supplementary figure 2.

A

hFlotillin-1 3'-UTR

```
5' ...GAUAACAAGACCUCAGUGCCUUC…………AAAAAAAUUGACUUCUCACCAGCU…3'
                          | | | | | | |                    | | |   | |         | | | | | | | |
            CCGUAAGUGGCGCACGGAAU        GCCGGACUAAGUGUU----GUGGUCGA
```

mir-124 **mir-138**

hCaveolin-1 3'-UTR

```
5' ...CUUGCCAAAUAGUUUGUGCCUUG…….. GCUGGCA----GUCCUGGAAGCCAGCU…3'
                           | | | | | | |           | | |          |   |         |       | | | | | |
             CCGUAAGUGGCGCACGGAAU        GCCGGACUAAGUGUUGUGGUCGA
```

mir-124 **mir-138**

B

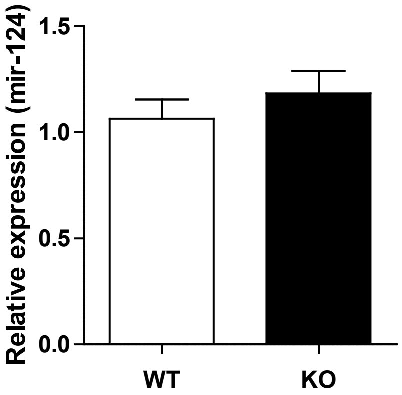

C

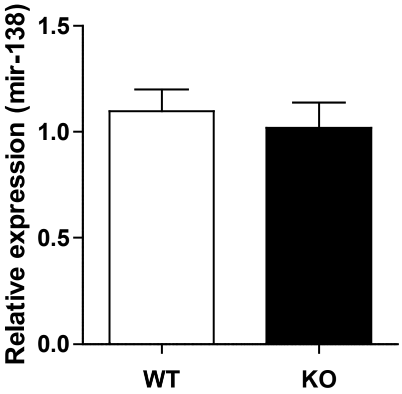

Supplementary figure 1 legend

Primary astrocytes from WT and DJ-1 KO mice were lysed using ice-cold 1% Triton X-100 buffer, and fractionated as described in 'Methods'. The soluble and insoluble fractions were analyzed for EAAT2 and DJ-1 by Western blotting.

Supplementary figure 2 legend

(A) Human flot-1 and cav-1 were predicted to be a target of miR-124 and -138 by PITA. (B) The level of miR-124 and -138 in WT and DJ-1 KO astrocytes were analyzed by quantitative RT-PCR.

Methods for supplementary figure 2

Total RNA was isolated from the cells using an miRNeasy Mini Kit (Qiagen), according to the manufacturer's instruction. Subseqently, cDNA synthesis and quantitative real-time PCR for miRNA was performed using miScript PCR Starter Kit (Qiagen) containing a miScript Reverse Transcriptase Mix, QuantiTect SYBR Green PCR Master Mix, and Universal primers, on on Rotor-Gene cyclers (Corbett Research), according to the manufacturer's instruction. The specific primers for miR-124, -138, and RNU as a loading control, were purchased from Qiagen. The values of miRNA were calculated using the delta Ct method and expressed as a change relative to the expression of RNU as an internal control.
